# Human CD4 T cells are a functional target for lipid nanoparticle-based mRNA vaccines

**DOI:** 10.1128/mbio.02254-25

**Published:** 2025-09-22

**Authors:** Samuel C. Kim, Jiin Felgner, Mariella S. Soto, Lauren Hitchcock, Emily K. Silzel, Haven Beares, Michael J. Hwang, Timothy B. Yates, Siyuan Cheng, Erika M. Joloya, Suhas Sureshchandra, Andrew M. Sorn, Zachary W. Wagoner, Delia Tifrea, Mustafa Kabeer, Douglas Trask, Shivashankar Othy, Philip Felgner, Lisa E. Wagar

**Affiliations:** 1 Department of Physiology & Biophysics, University of California Irvine 8788, Irvine, California, USA; 2 Institute for Immunology, University of California Irvine 8788, Irvine, California, USA; 3 Center for Virus Research, University of California Irvine 8788, Irvine, California, USA; 4 Adeline Yen Mah Vaccine Center, University of California Irvine 8788, Irvine, California, USA; 5 Division of Infectious Diseases, University of California Irvine 8788, Irvine, California, USA; 6 Department of Pathology and Laboratory Medicine, University of California Irvine 8788, Orange, California, USA; 7 Division of Pediatric Surgery, Children’s Hospital of Orange County, Orange, California, USA; 8 Department of Otolaryngology-Head and Neck Surgery, University of California Irvine 8788, Orange, California, USA; National Institute of Allergy and Infectious Diseases, Bethesda, Maryland, USA

**Keywords:** mRNA vaccines, adaptive immunology, translational immunology, lipid nanoparticles

## Abstract

Billions of mRNA-based severe acute respiratory syndrome coronavirus 2 (SARS-CoV-2) lipid nanoparticle (LNP) vaccine doses have been administered globally and shown to be both safe and effective. However, the mechanisms by which mRNA vaccines facilitate protection remain unclear. Peripheral blood analyses from vaccinated individuals show B and T cell responses are elicited, but it remains unknown which cells contribute to particle uptake and protein expression to elicit protective immunity. Using mRNA LNP fluorescent reporters, we show that CD4 T cells are an unexpected target for LNP-based protein expression *in vitro* and *in vivo*. CD4 T cells transfected with commercial SARS-CoV-2 mRNA vaccines are sufficient as the sole antigen producer to generate specific antibody responses but cannot support antigen presentation to other CD4 T cells. This study demonstrates that non-antigen-presenting immune cells are a novel target for mRNA-based protein expression and can support effective adaptive immune responses.

**IMPORTANCE:**

In this work, we demonstrate that CD4 T cells are a major target for lipid nanoparticle (LNP) transfection *in vitro* and *in vivo*. Using a human lymph node-like organoid model, we show that CD4 T cells effectively express the corresponding protein and support a productive humoral antigen-specific vaccine response to severe acute respiratory syndrome coronavirus 2 mRNA LNPs. Similarly, lymph node CD4 T cells are a major transfection target *in vivo* in a murine immunization model. Overall, we conclude that cells outside of the injection site can be significant contributors to the pool of antigen production after mRNA LNP immunization. This discovery opens the door to new vaccination strategies that could target CD4 T cells directly to optimize immune responses or immunotherapies.

## INTRODUCTION

Lipid nanoparticle (LNP)-based mRNA vaccines are immunogenic and highly effective for eliciting protective immunity to severe acute respiratory syndrome coronavirus 2 (SARS-CoV-2) (1–4). LNP-based formulations also hold many potential advantages over previous vaccine formats (5), including efficient manufacturing and the ability to rapidly update antigen sequences after mutation events. Despite their successes, the specific mechanisms by which mRNA vaccines elicit protective immunity are ambiguous, and multiple hypotheses have been proposed to explain how they confer immunogenicity and protection. Although LNPs are readily detected at the injection site after intramuscular delivery (6), the relative role of transfection and protein expression within the muscle or at other bodily sites, such as the draining lymph node (dLN), remains unclear. *In vitro* and *in vivo* experiments have shown that mRNAs delivered via lipid carriers can be sequestered by antigen-presenting cells (7–10), which subsequently express the encoded protein, and in some cases, present peptides to elicit antigen-specific responses. However, this finding does not preclude the possibility that additional cell types are also transfected and contribute to the immune response. Notably, murine and non-human primate (NHP) studies have shown that LNPs can traffic to distal sites, including the spleen and lymph nodes (6, 10), where LNPs have been detected in both lymphocytes and myeloid cells (6). Although these studies provided key insights into LNP distribution and uptake across various immune and non-immune tissues, most were of short duration (1–3 days). In contrast, SARS-CoV-2 mRNA-based LNPs support active germinal center (GC) responses for weeks to months post-vaccination (11), suggesting antigen is retained for extended periods in the lymphoid tissues. Thus, it is critical to elucidate the mechanisms and kinetics of LNP and immune cell interactions, protein expression, cell tropism, and bodily distribution to better understand how these factors contribute to vaccine immunogenicity.

In this study, we sought to determine which cells within human and murine lymphoid tissues can be transfected by LNPs and translate their cargo into functional protein, and whether these cells contribute to adaptive immunity. Using reporter LNPs formulated to mimic commercial mRNA vaccines, we show that CD4 T cells are efficiently transfected and represent the dominant protein producers among primary human lymphoid tissue cells. Using a commercial SARS-CoV-2 mRNA vaccine, we demonstrate that LNP uptake by cells exclusively in lymphoid tissues, and specifically from CD4 T cells, is sufficient to support a vaccine-specific antibody response.

## RESULTS

### LNP uptake and expression are dominated by CD4 T cells in primary human lymphoid tissues

**Fig 1:**
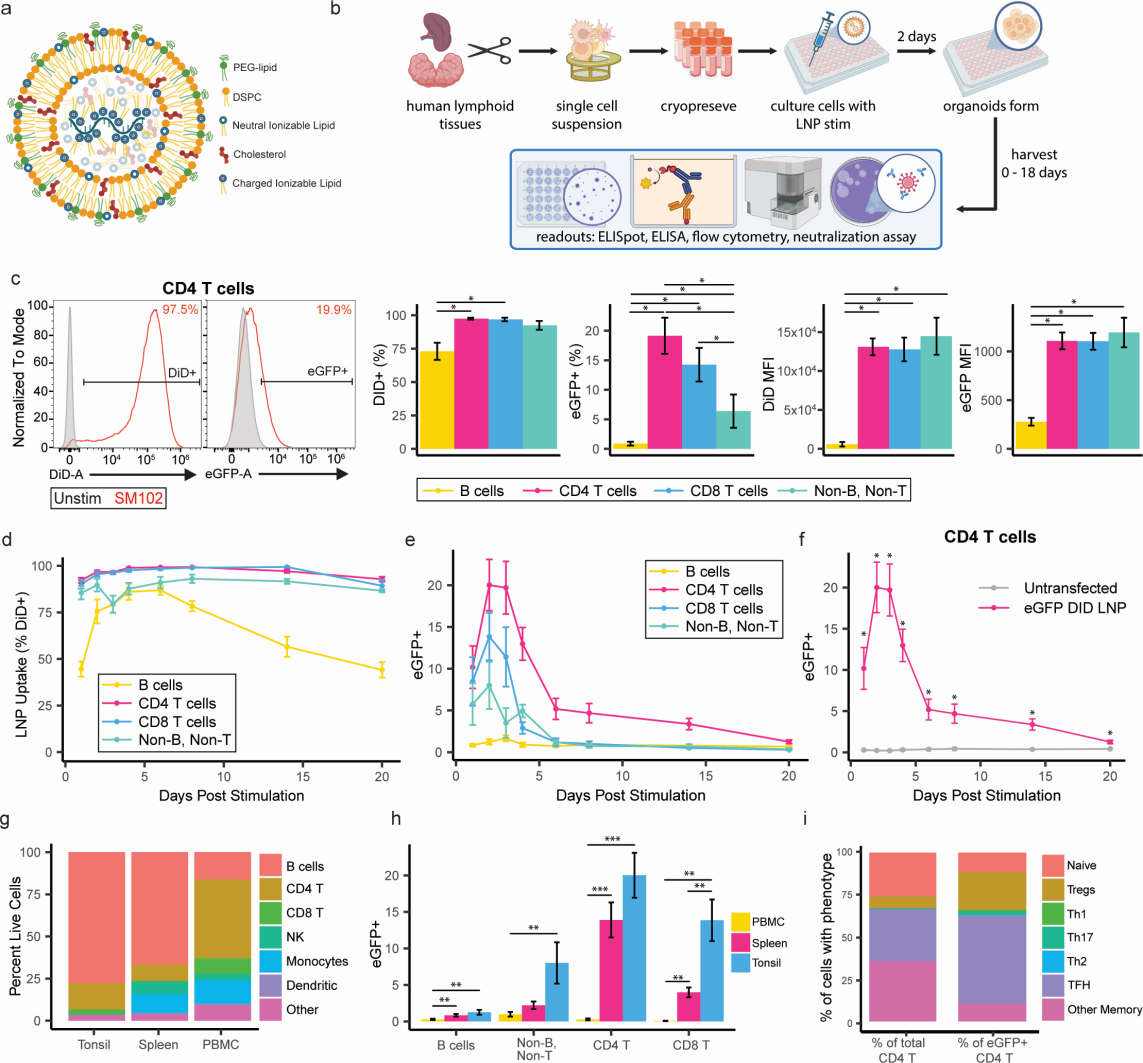
eGFP LNP transfection efficiency and protein expression in primary human lymphocytes. (**a**) Schematic describing general lipid nanoparticle structure. Created with BioRender. (**b**) Workflow for tissue processing, organoid culture preparation, and readouts of response to LNP treatment. Created with BioRender. (**c**) Particle uptake and protein expression following transfection of primary human B cells, CD4 T cells, CD8 T cells, and Non-B, Non-T cells with 0.5 µg of SM-102 eGFP DiD LNPs. Representative histograms shown are from tonsil organoids 2 days post-transfection from a representative donor. Summary data represent the frequency and fluorescence intensity of DiD+ and eGFP+ cells. Plotted values are medians ± s.e.m. (*n* = 6). (**d**) Longitudinal analysis of tonsil organoids after stimulation with SM-102 LNPs for LNP uptake in major cell subsets. (**e**) mRNA translation into protein in major cell subsets. (**f**) Statistical analysis of eGFP expression in CD4 T cells from transfected vs. untransfected tonsil organoids (*n* = 6). Plotted values represent means ± s.e.m. (**g**) Cell composition of human tonsil, spleen, and blood-derived cells *ex vivo*. (**h**) mRNA translation (as measured by eGFP) in primary human peripheral blood mononuclear cells (PBMCs; *n* = 9), spleen (*n* = 6), and tonsil cultures (*n* = 6) after stimulation with SM-102 eGFP LNPs. Plotted values represent the mean ± s.e.m. (**i**) Phenotypes of total (left) and eGFP+ (right) CD4 T cells (*n* = 4). Plotted values are medians ± s.e.m. *P < 0.05; **P < 0.01; ***P < 0.001, ****P < 0.0001.

To understand LNP transfection efficiency, we first generated an enhanced green fluorescent protein (eGFP) mRNA reporter LNP to probe transfection efficiency, translation, and protein expression in cell lines, primary cells from human lymphoid tissues and mice. The general LNP design is shown in Fig. 1a. LNPs were prepared in-house using 9-heptadecanyl 8-{(2-hydroxyethyl)[6-oxo-6-(undecyloxy)hexyl]amino}octanoate (SM-102), along with co-lipids distearoylphosphatidylcholine (DSPC), cholesterol, and 1,2-dimyristoyl-rac-glycero-3-methoxypolyethylene glycol-2000 (DMG-PEG 2000), with a molar ratio of 50/10/38.5/1.5 to encapsulate mRNA (Fig. S1a). This formulation is identical in lipid composition to commercial SARS-CoV-2 mRNA vaccines developed by Moderna. The resulting particle size distribution of SM-102 LNPs was monodisperse with a hydrodynamic diameter of 96 nm, as measured by dynamic light scattering (Fig. S1b). To track cell interactions with LNPs and transfection and translation efficiency, SM-102 particles were prepared with a trace amount of lipophilic fluorescent dye, 1,1-dioctadecyl-3,3,3,3-tetramethylindodicarbocyanine (DiD), and loaded with mRNA encoding eGFP. Encapsulation of eGFP mRNA (93% by Ribogreen assay) was highly efficient (Fig. S1c), and transfection into three cell lines (HEK293T, Jurkat, and K562) showed highly efficient LNP uptake and eGFP expression (>90%) by 24 hours post-transfection (Fig. S1d), indicating LNPs were of high quality.

Having established the functional quality of the particles, we assessed LNP uptake and protein expression in cells from primary human lymphoid tissues. Tissues were processed as previously described (12) into single-cell suspensions and cultured into organoids to investigate the kinetics of LNP uptake and protein expression in primary human immune cells (Fig. 1b for organoid workflow). LNPs were efficiently taken up, as indicated by high DiD fluorescence intensity, in CD4 and CD8 T cells (Fig. 1c for representative donor and summary data). Particles were significantly less efficiently taken up by B cells compared to CD4 and CD8 T cells, as measured by the frequency and median fluorescence intensity of DiD +cells (Fig. 1c).

Given our initial observations that LNP interaction efficiency and protein translation depended on cell identity, we considered whether this disparity could be explained by cell type-specific differences in the kinetics of transfection and translation. Previous work showed cells take up LNPs within hours, express the corresponding protein, and decline in expression within 5 days (13–15). However, spike protein has been observed in axillary lymph nodes at least 60 days post-vaccination (16). To reconcile these findings, we profiled the kinetics of particle uptake and protein expression among different immune cell subsets in tonsil organoids (12). Although most organoid cells interacted with LNPs, the kinetics of LNP uptake, protein expression, and decline was far slower than has been reported from conventional cell line analyses. In tonsil organoids, cells reached maximum DiD positivity by day 3, with >95% of cells in culture having interacted with LNPs (Fig. 1d). To our surprise, over 95% of CD4 T cells and CD8 T cells remained DiD+ for at least 20 days, while B cells had a bigger drop compared to peak uptake, with about ~45% remaining DiD+ by day 20 (Fig. 1d). In line with the kinetics of LNP uptake, eGFP+ cells appeared within the first 2 days of culture (Fig. 1e). CD4 T cells were the dominant protein-expressing population in organoids, with peak expression (15%–25% eGFP+) detected from days 2 to 4 in culture and gradually declining thereafter. However, a small proportion of CD4 T cells continued to express eGFP for up to 20 days after LNP treatment (Fig. 1f). Although LNPs were efficiently taken up by organoid B cells, CD8 T cells, and non-B and non-T cells, no eGFP expression was detected in these populations after day 6.

Having shown that tonsil organoids could express eGFP encoded by mRNA LNPs, we asked whether LNP-associated protein translation and expression were influenced by cell composition. To do this, we analyzed LNP uptake across human tonsils, spleen, and peripheral blood mononuclear cells (PBMCs), which have diverse immune compositions. As expected, *ex vivo* analysis of representative samples showed proportionally more monocyte and dendritic cell representation in spleen and PBMCs compared to tonsils (Fig. 1g). Cells isolated from each source were treated with eGFP mRNA LNPs and analyzed 2 days post-transfection. Despite increased APC representation in human splenocytes compared to tonsils, spleen-derived CD4 T cells still took up and expressed eGFP LNPs (Fig. 1h), similar to levels in tonsils. eGFP mRNA LNP-stimulated PBMC cultures did not express substantial amounts of eGFP in any cell type (Fig. 1h).

Given that CD4 T cells were elite producers of the LNP-encoded protein in both spleen and tonsil tissues, we further investigated the relationship between LNP uptake and CD4 T cell phenotype. Among tonsillar eGFP+ CD4 T cells, which represented approximately 18% of total CD4 T cells on day 2 post-LNP treatment, most were of a memory phenotype (Fig. 1i), with enrichment in eGFP+ regulatory (Tregs) (7%–22%) and follicular helper (TFH) T cells (31%–54%) compared to their baseline proportions. Based on these data, we conclude that the kinetics of particle uptake and protein expression differ substantially in primary human lymphoid tissue cells compared to cell lines, with primary lymphocytes showing lower transfection efficiency and delayed kinetics in LNP uptake but prolonged protein expression.

### CD4 T cells are a dominant LNP target in the dLN *in vivo*

**Fig 2:**
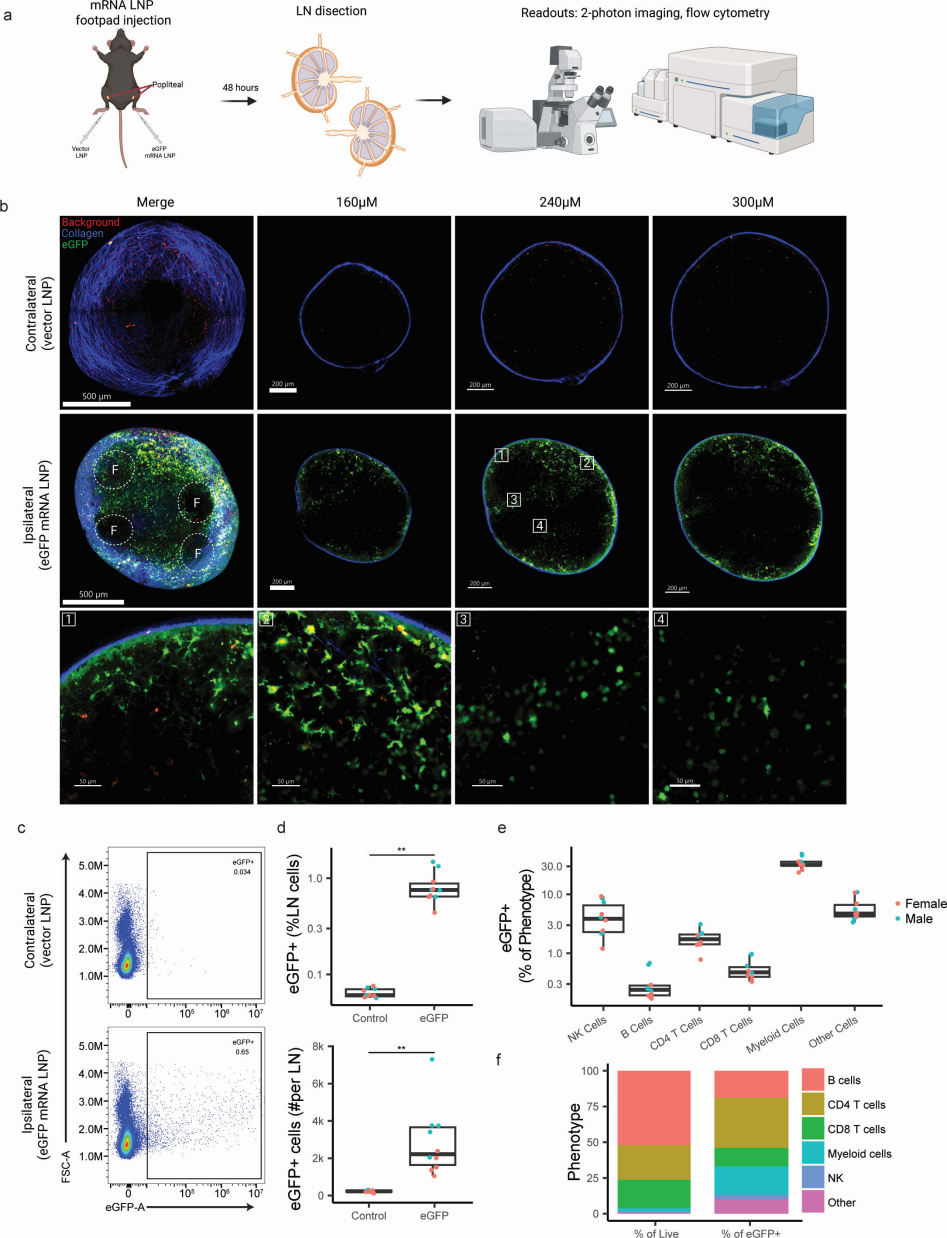
eGFP LNP transfection of popliteal lymph nodes following injection of murine foot pad. (**a**) Workflow of footpad injection and popliteal lymph node analysis. Created with BioRender. (**b**) Representative 2-photon images of popliteal lymph nodes, with contralateral vector LNP (top), ipsilateral eGFP mRNA LNP (middle), and magnified regions from the ipsilateral eGFP mRNA LNP lymph node at 240 µm depth (bottom) at 48 hours post-injection (*n* = 3). B cell follicles are outlined and labeled as “F.” (**c**) Representative plot of contralateral (top) and ipsilateral (bottom) lymph nodes. (**d**) Proportion and count of eGFP+ lymph node cells (*n* = 10). (**e**) Percentage eGFP+ of individual cell phenotypes (*n* = 10). (**f**) Phenotypes of total (left) and eGFP+ (right) ipsilateral lymph node cells (*n* = 10). Plotted values are means ± s.e.m. *P < 0.05; **P < 0.01; ***P < 0.001, ****P < 0.0001.

Although *in vitro* models are valuable for gaining insights into LNP and cell interaction dynamics, differences in LNP delivery and distribution could lead to distinct outcomes compared to *in vivo*. Therefore, we analyzed reporter distribution patterns following *in vivo* vaccination in mice following LNP injection into rear foot pads (Fig. 2a for workflow). Two-photon imaging of popliteal lymph nodes revealed eGFP+ signal (~300 µm depth) selectively in the ipsilateral side within 48 hours of injection (Fig. 2b). eGFP was readily detected in the subcapsular zone (Fig. 2b, regions 1 and 2), likely representing subcapsular sinus macrophages, but also in the interfollicular regions surrounding the B cell follicles (Fig. 2b, regions 3 and 4). Flow cytometry analysis of dissociated lymph nodes showed approximately 1% of cells in the ipsilateral lymph node expressed eGFP, with typically 2,100 GFP+ cells per dLN (Fig. 2c and d). eGFP analysis for each major cell type revealed that transfection and protein expression efficiency were highest in myeloid cells, with about a third of myeloid cells becoming eGFP+ (Fig. 2e) and lower frequencies among lymphocytes. However, phenotypic analysis of eGFP+ cells showed, similar to our human tonsil organoid observations, one-third of the total eGFP+ cells were CD4 T cells (Fig. 2f). Overall, we conclude that due to their abundance in lymphoid tissues, CD4 T cells represent a non-trivial proportion of the protein-producing cells in the lymph node following mRNA LNP immunization *in vitro* and *in vivo*.

### Human immune organoids produce antigen-specific responses to SARS-CoV-2 LNPs

**Fig 3:**
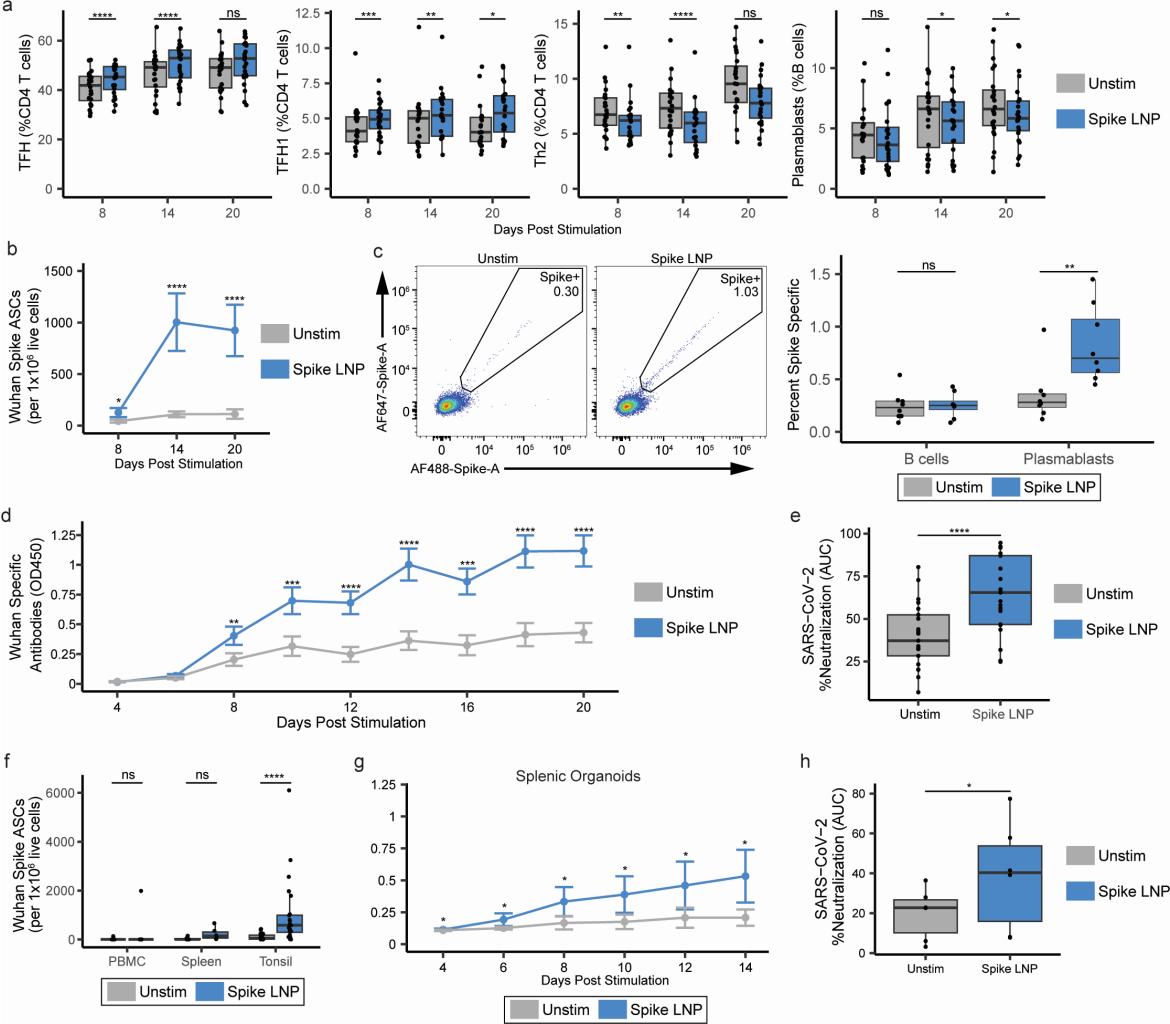
Lymphoid organoid responses to SM102-based SARS-CoV-2 spike LNP vaccine. (**a**) Tonsil organoids (*n* = 24) were stimulated for up to 20 days with 0.5 µg of SARS-CoV-2 mRNA vaccine and harvested at various time points post-treatment to assess the developing B cell, T cell, and antibody response. Cell frequencies were assessed by flow cytometry on days 8, 14, and 20 post-stimulation. Flow gating strategy depicted in Fig. S2a through c. (**b**) SARS-CoV-2 spike-specific antibody-secreting cells (ASCs) from tonsil organoids. (**c**) Representative flow cytometry staining and summary data of SARS-CoV-2 spike-specific B cells and plasmablasts from day 14 tonsil organoids. (**d**) Kinetics of secreted antibodies specific for SARS-CoV-2 (Wuhan) spike protein. (**e**) Virus neutralization of SARS-CoV-2 (Washington strain) by tonsil organoid culture supernatants containing spike-specific antibodies. Neutralization values represent the area under the curve (AUC) of serially diluted culture supernatants (*n* = 21). (**f**) SARS-CoV-2 spike-specific antibody-secreting cells from tonsils (*n* = 24), spleen (*n* = 6), and PBMCs (*n* = 10). (**g**) Magnitude and (**h**) virus neutralization of antibodies secreted from SARS-CoV-2 LNP-treated spleen organoids (*n* = 6). Box plots show median values with hinges representing the first and third quartiles and whiskers representing the highest and lowest value within 1.5 times the interquartile range of the hinges. *P < 0.05; **P < 0.01; ***P < 0.001, ****P < 0.0001.

Having shown that lymphoid tissue cells can directly express proteins encoded by mRNA LNPs, we evaluated their potential to boost adaptive immune responses to a vaccine-relevant antigen. To achieve this, we treated tonsil organoids with commercial SARS-CoV-2 mRNA LNPs and tracked changes in total and antigen-specific B cell responses and T cell phenotype changes for up to 20 days (gating strategy depicted in Fig. S2a through c). Organoids were generated from tissue specimens collected throughout the COVID-19 pandemic; we expect most donors were previously exposed to SARS-CoV-2 antigens, either through vaccination or infection. Organoids from both control and SARS-CoV-2 LNP conditions were viable (88% vs. 86% on day 8; Fig. S3a). Total TFH, and particularly TFH with a Th1-like phenotype, were consistently elevated in SARS-CoV-2 LNP-treated organoids compared to untreated controls (Fig. 3a). In contrast, Treg and Th1 proportions were unchanged by LNP treatment, while Th2 cells decreased (Fig. 3a; Fig. S3a).

We next analyzed changes in the B cell population. Although LNP treatment led to slight declines in overall B cell differentiation into plasmablasts, pre-GC, and GC B cells (Fig. 3a; Fig. S3a), SARS-CoV-2-specific antibody-secreting cells (ASCs) significantly increased following LNP treatment (Fig. 3b). ASCs remained at peak levels (median 583 per 10^6^ live cells) from days 14 to 20 post-stimulation. Analysis of SARS-CoV-2 spike-specific B cells (through direct antigen labeling) showed LNP treatment significantly enhanced antigen-specific plasmablast differentiation, while the total pool of antigen-specific B cells remained unchanged (Fig. 3c). Finally, we assessed the magnitude and quality of the specific antibody response. In contrast to our previous studies with inactivated influenza vaccines, where most organoid antibody responses peaked 7–10 days following antigen stimulation (17), spike-specific antibodies were first detected 8 days post-stimulation and continued to increase until the conclusion of the culture on day 20 (Fig. 3d). Antibodies generated by vaccine-stimulated organoids were functional and could neutralize SARS-CoV-2 2020 Washington (WA1) wild-type virus (Fig. 3e). To rule out non-specific activation due to the mRNA LNP themselves, we stimulated cultures with a scrambled mRNA LNP with and without the addition of recombinant spike protein (Fig. S3b). We observed no significant differences in SARS-CoV-2-specific ASCs between unstimulated, scrambled LNPs alone, and scrambled LNPs plus recombinant spike protein, suggesting that stimulation with LNPs alone does not induce an antigen-specific response (Fig. S3b).

To investigate the capability of other immune sites to respond to SARS-CoV-2 LNPs, organoids were prepared from human splenocytes or PBMCs. PBMCs did not support SARS-CoV-2-specific B cells or antibody responses except in one donor, likely due to recent infection or vaccination (Fig. 3f). Compared to lymphoid tissue-derived organoids, PBMC-derived T cells showed limited (but statistically significant) increases in LNP treatment-associated TFH differentiation (Fig. S3c). Spleen organoids were responsive to SARS-CoV-2 LNPs, and like tonsils, they did not show a significant change in the total frequency of plasmablasts (Fig. S3c) but did support SARS-CoV-2-specific ASC differentiation (Fig. 3f) and neutralizing antibody secretion with similar kinetics to tonsil organoids (Fig. 3g and h). Interestingly, ASC responses in splenic organoids were notably lower than those of tonsil organoids. These findings show that SARS-CoV-2 LNP transfection of lymphoid tissue cells, but not peripheral blood immune cells, is sufficient to elicit SARS-CoV-2 ASCs and stimulate neutralizing antibody production, indicating the composition of the lymphoid tissue microenvironment is critical for supporting SARS-CoV-2 LNP antibody responses.

### LNP transfection of CD4 T cells is sufficient to support antibody but not CD4 T cell responses to SARS-CoV-2 mRNA vaccines

**Fig 4:**
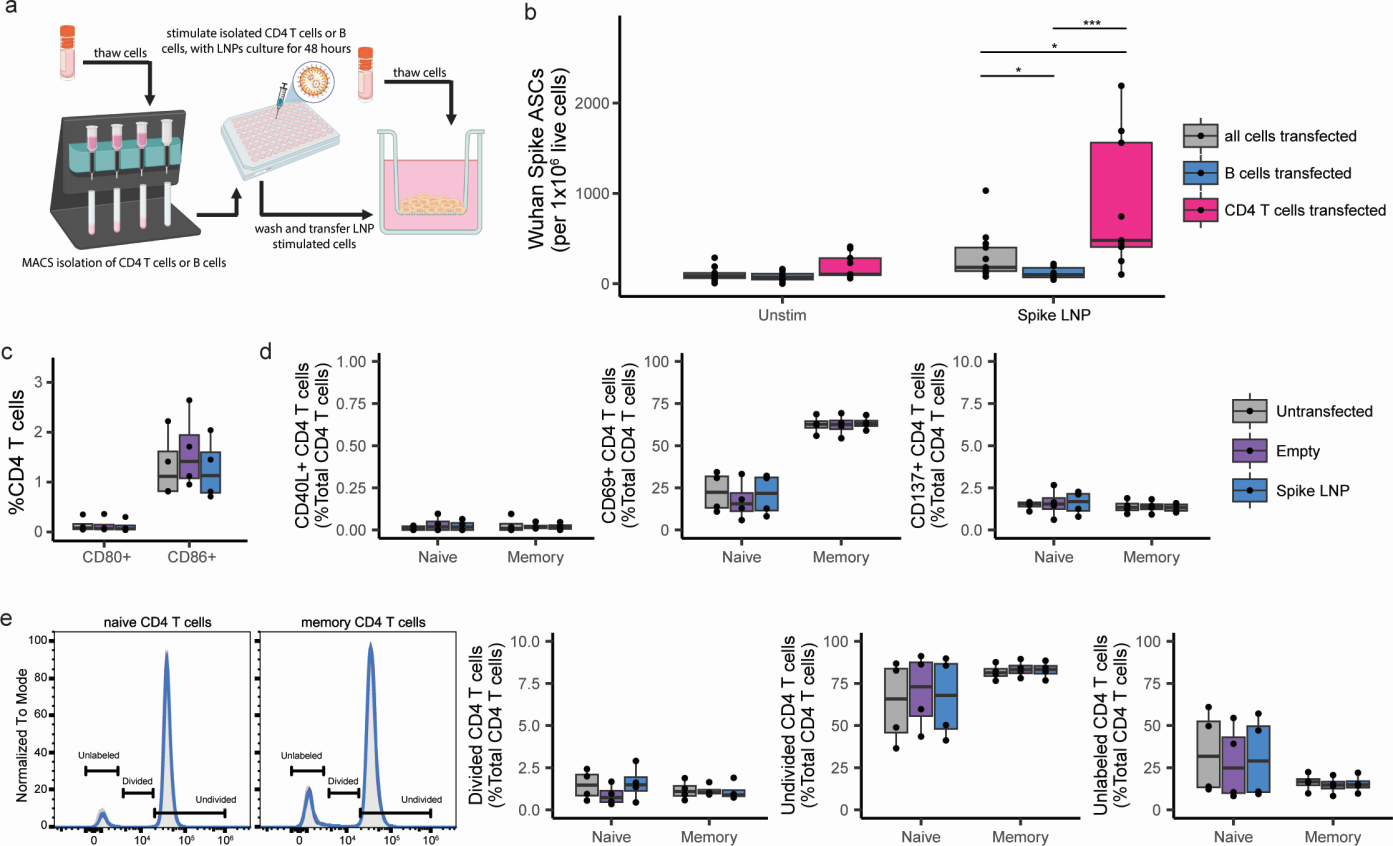
CD4 T cell SARS-CoV-2 LNP vaccine transfection. (**a**) Workflow for isolation and transfection of CD4 T or B cells. Created with BioRender. (**b**) SARS-CoV-2 ASCs from unmanipulated tonsil organoids (*n* = 13), tonsil organoids with B cells transfected in isolation (*n* = 10), or tonsil organoids with CD4 T cells transfected in isolation (*n* = 8). (**c**) Flow cytometry analysis of CD4 T cells 2 days following transfection with SARS-CoV-2 LNPs (*n* = 4). (**d**) Flow cytometry analysis of naive and memory CD4 T cells co-cultured with transfected CD4 T cells 7 days post co-culture (*n* = 4). (**e**) Representative histograms of CD4 T cell divisions 7 days post-co-culture (left) and quantification of divided, undivided, and unlabeled CD4 T cells (right; *n* = 4). Box plots show median values with hinges representing the first and third quartiles and whiskers representing the highest and lowest value within 1.5 times the interquartile range of the hinges. *P < 0.05; **P < 0.01; ***P < 0.001; ****P < 0.0001.

We showed that CD4 T cells are elite protein producers following LNP treatment, and increasing the number of APCs, through the use of splenocytes rather than tonsils, did not enhance antibody responses. Therefore, we hypothesized that CD4 T cells as the sole source of mRNA LNP protein may be sufficient to support at least some elements of the adaptive immune response. To address this question, isolated tonsillar CD4 T cells were transfected with SARS-CoV-2 LNPs, rested for 48 hours, thoroughly washed, then transferred into autologous tonsil organoid cultures (Fig. 4a). Organoids where the only source of LNP-treated cells was from the CD4 compartment produced significantly better ASC responses compared to organoids where SARS-CoV-2 LNPs were added to all cells (Fig. 4b). An analogous experiment, where B cells were isolated and independently transfected, did not induce a SARS-CoV-2 antibody response (Fig. 4b). This finding suggests that protein production from CD4 T cells alone is sufficient to support differentiation of ASCs in response to SARS-CoV-2 mRNA vaccines.

We next asked whether transfected CD4 T cells could also serve as professional APCs to other CD4 T cells. To test this, we isolated and transfected CD4 T cells (tCD4 T cells), cultured them for 2 days, washed them, then added them to autologous, untransfected naive or memory CD4 T cells at a 1:10 ratio. Analysis of tCD4 T cells 2 days post-transfection showed no expression of costimulatory or activation markers associated with class II antigen presentation (Fig. 4c). No priming (Fig. 4d) or proliferation (Fig. 4e) of target CD4 T cells was observed in the co-cultured CD4 T cells. Based on these data, we conclude that although CD4 T cells are the dominant source of antigen post-mRNA LNP transfection, they are not able to prime or reactivate other CD4 T cells.

## DISCUSSION

In this study, we elucidated new mechanisms by which mRNA LNPs interact with and modulate immune responses directly in human lymphoid tissues. Our initial analyses indicated that CD4 T cells were the primary interacting cell type and could express proteins encoded by mRNA LNPs. The kinetics of protein expression was distinct from what has been observed in other cell types and cell lines, with sustained expression over several weeks. We also demonstrated that a commercial SARS-CoV-2 mRNA vaccine can support the development of a robust humoral immune response in lymphoid tissues, even in the absence of cells draining from peripheral sites. LNP transfection is exceedingly efficient in immortalized cells (14, 18), but transfection both *in vivo* and in primary *in vitro* culture is much less efficient, with additional complexity but more physiological relevance due to mixed cell populations. Furthermore, the kinetics of LNP uptake and protein translation was slower in primary lymphocytes than previously shown for cell lines and phagocytic primary cells.

CD4 T cells showed the highest proportion of eGFP+ cells, while smaller proportions of CD8 T cells and non-B, non-T cells also expressed the protein. Intriguingly, protein production from CD4 T cells alone was sufficient to support robust humoral responses to a SARS-CoV-2 mRNA vaccine. However, transfected CD4 T cells were unable to induce CD4 T cell priming or recall, implying that they function as a source of protein but do not process and present to antigen-specific T cells in isolation. This suggests that observed responses in the lymphoid organoid system to spike mRNA LNP vaccine stimulation rely on recall responses rather than on generating primary responses. Interestingly, high LNP uptake did not always correlate with protein expression. B cells did not support protein translation, and transfection of isolated B cells did not induce antigen-specific antibody responses, suggesting that even similar cell types have different mechanisms of endosomal escape (19, 20) that ultimately affect protein translation. Although SM-102-based vaccines are clearly effective *in vivo* (21, 22, 22–24), future work to optimize LNP lipid composition to efficiently target desired cell types could enhance particle uptake and translation.

Although APCs at the injection site likely play an important role in initiating the antigen-specific response *in vivo*, a substantial quantity of injected LNPs drain to the lymph nodes (6) and thus may act directly on lymphoid tissue cells. *In vivo*, we confirmed that lymph node cells take up and express LNPs, with primarily CD4 T cells and APCs as the targets. APCs such as dendritic cells, monocytes, and macrophages play an essential role in LNP uptake and protein expression (7–10, 25). However, the use of spleen organoids and PBMC cultures, which contain substantially more APCs than tonsil organoids, did not improve SARS-CoV-2 antibody responses. Although these data do not preclude a role for APCs in initiating and facilitating the adaptive immune response to LNPs, they do suggest that APCs are not required to be the direct producers of the mRNA-encoded antigen.

Of relevance for vaccine design, we showed that TFH in lymphoid tissues are amenable to LNP uptake and protein production and could sustain expression for at least 2 weeks. Antigen production by TFH may improve protein accessibility to B cells directly in the lymph node without reliance on antigen draining from distal sites or immune complex deposition on follicular dendritic cells. Furthermore, in lymphoid tissue organoids stimulated with commercial mRNA spike vaccine, we observed an increase in TFH and acquisition of a Th1-like phenotype. This finding is consistent with prior reports showing LNP-mediated TFH differentiation in an antigen-agnostic manner in vaccinated animals (26), increases in peripheral blood circulating TFH transiently following vaccination in humans (27, 28), and TFH expansion post-vaccination in human lymph node fine-needle aspirates (29). Although a mechanism has not yet been described, TFH differentiation following mRNA vaccination can be desirable for eliciting robust responses (30–32). Combined with our finding that TFH are a major target for mRNA LNPs, we speculate that LNPs may facilitate enhanced B cell help through antigen expression and availability on TFH.

A vaccine design that supports a prolonged GC response is highly beneficial for inducing increased antibody magnitude, breadth, and quality (16, 31, 33). We detected prolonged humoral antigen-specific responses in LNP-treated organoids (17), in contrast to the relatively short-lived responses we observe with non-LNP vaccine formats in immune organoids. We speculate that the extended immune activity following LNP treatment may be due to sustained antigen production, in part from CD4 T cells, and could explain the longer-lived GC response following SARS-CoV-2 mRNA vaccination *in vivo* (16, 33). Based on the pattern and persistence of protein expression from our reporter LNP studies and functional capacity to support an antigen-specific humoral immune response, we postulate that CD4 T cells represent an important and unexpected source of antigen production for B cells following mRNA vaccination.

## MATERIALS AND METHODS

### Informed consent and sample collection

Tonsils were procured from consented individuals undergoing surgery for obstructive sleep apnea, hypertrophy, or recurrent tonsillitis, or as discarded surgical materials. Participant tissues were collected in accordance with the University of California, Irvine Institutional Review Board (protocol 2020-6075 or through a non-human subjects determination). All participants provided written informed consent. Spleens from six individuals undergoing surgery were collected at the University of California, Irvine Medical Center or Children’s Hospital of Orange County as discarded surgical materials with approval by the UCI IRB. PBMC samples used in this study were purchased from AllCells. Please see Table S1 for participant-level demographic information.

### Tissue collection and processing

Samples were processed as previously described (17). Whole tonsils were collected immediately following surgery and immersed in an antimicrobial bath of Ham’s F12 medium (Gibco) and penicillin-streptomycin (Gibco) for 30–60 min at 4°C for decontamination of the tissue. Tonsils were then washed with phosphate-buffered saline (PBS) and mechanically dissociated; debris was removed using gradient centrifugation (Lymphoprep, Stemcell). Samples were cryopreserved in fetal bovine serum (FBS) with 10% dimethyl sulfoxide (DMSO) and stored in nitrogen until use.

Splenic samples were collected from pathology in Ham’s F12 medium (Gibco). The splenic capsule was manually removed, the tissue was cut into small pieces with scissors, and then mechanically dissociated to a single cell suspension. Red blood cells were lysed with RBC lysis buffer (Biolegend). Dead cells and debris were removed by density gradient centrifugation (Lymphoprep, Stemcell). Samples were cryopreserved in FBS with 10% DMSO and stored in nitrogen until use. PBMCs were procured as cryopreserved samples from AllCells.

### Encapsulation of mRNA in LNPs

LNPs were formulated with ionizable lipid SM-102 (Broadpharm, cat# BP-25499) along with other co-lipids DSPC (Avanti Polar Lipids, cat# 850365), cholesterol (Avanti Polar Lipids, cat# 700100), and PEG2000-DMG (Avanti Polar Lipids, cat# 880151) with a molar composition of 50/10/38.5/1.5. Their chemical structures are shown in Fig. S1a. To label LNPs, lipophilic DiD (Invitrogen, cat# D7757) was added at a composition of 0.2 mole%. eGFP mRNA and spike mRNA were obtained from Tri-Link and OZ Biosciences, respectively.

Lipids were dissolved in ethanol and rapidly combined with mRNA in a 100 mM sodium acetate (NaOAc) buffer, pH 4, at a volume ratio of 1:3 (ethanol) and an N/P ratio of 6 (commonly known as the charge ratio of cationic lipid to the negatively charged mRNA). The combination was performed by microfluidic mixing using Precision NanoSystem’s “Ignite” (Precision NanoSystems, Vancouver, BC, Canada) or rapid mixing via T-junction using a dual syringe pump. The ionizable lipid became protonated at low pH and electrostatically bound to the anionic phosphate backbone of the mRNA, driving vesicle formation and mRNA encapsulation. The pH was then raised to neutral by dialysis with over 100 volumes of 20 mM Tris, 4.3 mM sodium acetate, pH 7.4, 10% (wt/vol) sucrose (TAS buffer) for at least 5 hours to form neutral LNPs with ethanol simultaneously removed. Finished mRNA LNPs were further concentrated using a centrifugal ultrafilter (Amicon Ultra-4, MWCO = 10 kDa) if needed. The resulting LNPs were characterized for (i) particle size and polydispersity index (PDI) by dynamic light scattering using a Malvern Zetasizer, (ii) mRNA encapsulation by RiboGreen assay in the absence and presence of Triton X-100, and (iii) functional protein expression by *in vitro* transfection. The commercial SARS-CoV-2 mRNA LNP vaccine was used for comparative evaluation.

### Cell line transfection

HEK293T, Jurkat, and K562 cell lines were sourced from ATCC. Cells were cultured in Dulbecco’s modified Eagle medium (DMEM) +10% FBS, 1× sodium pyruvate, and 1× Antibiotic-Antimycotic (Gibco). Cells were seeded in tissue culture plates at a concentration of 7.5 × 10^5^ cells per well in 1 mL final volume and treated with 2 µg/mL LNPs.

### Organoid preparation

To generate organoids, cryopreserved cells were thawed and then plated at a final density of 7.5 × 10^6^ /mL in 200 µL final volume in 96-well ultra-low attachment plates (Corning) or at 6 × 10^6^ cells per transwell (Corning) in 1 mL final volume in a 12-well tissue culture plate as previously described (12, 27). Organoid media was composed of RPMI1640 with Glutamax, 10% FBS, 1× non-essential amino acids, 1× sodium pyruvate, 1× Antibiotic-Antimycotic (Gibco), 1× Normocin (InvivoGen), 1× insulin, selenium, transferrin supplement (Gibco), and 0.5 µg/mL recombinant human BAFF (Biolegend). LNPs were used at a dose of 0.5 µg per well for 96-well plate cultures and 2 µg per well for transwell cultures. Cultures were incubated at 37°C, 5% CO_2_ with humidity, and the media was replenished every other day by exchanging 33% of the volume with fresh organoid media.

### Flow cytometry

Immune organoids were harvested, and cells were washed with fluorescence-activated cell sorting buffer (FACS) buffer (PBS with 3% FBS, 0.05% sodium azide, and 2 mM EDTA) to remove any residual antibodies or factors generated during culture. All samples were first stained using fixable viability dye (Zombie Aqua, Biolegend) in PBS for 15 minutes on ice with Fc blocking. Following washes, cells were stained using antibody cocktails prepared in FACS buffer for 30 minutes on ice while protected from light. Data were collected using a Cytek Aurora Spectral Flow Cytometer. Cell sorting was performed using a BD FACSAria Fusion instrument.

### Antibody detection by ELISA

SARS-CoV-2 Spike-specific antibodies were detected by coating high-binding assay plates (Corning) with spike protein at a final concentration of 2.5 µg/mL in 100 mM sodium carbonate/bicarbonate ELISA coating buffer. Non-specific binding was blocked by incubating plates with 1% BSA (in PBS) for 2 hours at RT. Culture supernatants were diluted 1:5 with PBS and added to coated, blocked plates for 1 hour at RT. Horseradish peroxidase-conjugated anti-human secondary antibodies to IgM/IgG/IgA (Abcam) were used to detect bound antibodies. Plates were developed with the substrate and then stopped with 1N HCl. The optical density (A450) was reported as a semi-quantitative measure of Ab concentration.

### Neutralization

50,000 Vero E6 cells were seeded per well and incubated for 24 h. Supernatants from SARS-CoV-2 mRNA vaccine-stimulated and non-stimulated organoids were serially twofold diluted (to 1:32). Diluted supernatants were then incubated (in duplicate) with live Washington strain SARS-CoV-2 (Microbiologics: G2027B) at a multiplicity of infection of 0.01 (310,000 pfu/mL = 0.002 mL per well) for 30 minutes before the addition of VeroE6 cells; a virus-only control was also included. The sample/virus supernatant was co-incubated for 45 minutes with Vero E6 before removal, then the cells were incubated in 1% MTC (10% FBS, 1% PenStrep in DMEM) for 24 hours before fixation with 4% paraformaldehyde overnight. Plates were processed using N-specific primary antibody (Novus; NB100-56576) followed by HRP Donkey anti-Rabbit IgG secondary Antibody (BioLegend: 406401) and stained prior to imaging. Plaques were counted by hand after scanning the plate using an ELISpot reader. Neutralization was calculated using the following equation: Neutralization = 100 − (# plaques obtained with a given antibody concentration/# plaques with virus only) × 100.

### ELISpot

SARS-CoV-2 Spike-specific ASCs were detected using an ELISpot protocol as previously described with modifications (12). Organoids were harvested, and the cells were washed with ELISpot cell resuspension media, counted, then plated on SARS-CoV-2 Spike-coated and blocked 96-well PVDF membrane plates (Millipore). Each sample was plated in duplicate and with a total live cell count of 5 × 10^5^ cells per well. Cells were incubated on membranes undisturbed for 5 hours at 37°C. Plates were then washed and treated with horseradish peroxidase-conjugated anti-IgG/IgA/IgM secondary antibody (Abcam). After incubation overnight at 4°C, plates were washed and developed with AEC substrate (BD), washed 20 times with water, dried, and spots were enumerated (Immunospot S5 Core Analyzer).

### Statistical analysis

All statistical analyses were performed using R (R 4.3.1) (34, 35) and ggpubr. For comparing multiple paired groups, Wilcoxon signed-rank tests were used to identify significant differences. For comparing multiple unpaired groups, Wilcoxon rank-sum tests (Mann–Whitney U tests) were used to identify significant differences and corrected for multiple testing where relevant. *P* < 0.05; **P < 0.01; ***P < 0.001, ****P < 0.0001.

### Mice, mouse injections, and multiphoton imaging of eGFP+ cells in dLNs

All animal work was approved by the University of California, Irvine Institutional Animal Care and Use Committee Protocols (AUP-24-131 and AUP-21-133). The laboratory animal resources at UCI are internationally accredited by the Association for Assessment and Accreditation of Laboratory Animal Care (AAALAC #000238). All mice were housed in a clean, specific pathogen-free facility, and both male and female mice from the C57Bl/6 background were used at 8–12 weeks of age. C57BL/6 mice (Jackson Laboratory, Sacramento, CA) were subcutaneously injected with SM102 LNPs, containing either Spike mRNA (control for fluorescence) or eGFP mRNA into the rear footpads under deep surgical anesthesia (1.8% isoflurane). Two days after injection, mice were euthanized using CO_2_, and popliteal dLNs were dissected and attached to a plastic coverslip using Vetbond Tissue Adhesive (3M, Saint Paul, MN) for imaging as described previously (36). The explanted dLNs were imaged using a Leica Stellaris 8 DIVE FALCON multiphoton microscope fitted with a Leica 20× water-immersion objective (HC APO, NA = 1.0, WD = 1.95 mm). Chameleon Vision II laser was tuned to 920 nm for 2-photon excitation of eGFP. Spectral non-descanned detectors (HyD X) were used to collect images using the 4Tune setup. Second harmonic generation signal from collagen fibers in the lymph node capsule was detected at 450–470 nm, eGFP signal was collected from 490 to 560 nm, and autofluorescence signal was registered with detector bandwidth set to 600–640 nm. For three-dimensional montage imaging of the whole lymph node, blocks of images (voxel size: 0.541 μm × 0.541 µm × 4 µm) were acquired and stitched using Leica LAS X software. 3D images were analyzed using Imaris software (version 10.2.0) and processed with a median filter (size: 3 × 3 × 1) on all channels before exporting images and movies.

### Draining LN phenotyping using flow cytometry

Popliteal dLNs were isolated and homogenized using a BioMasher II (RPI, CAT# 199623) and digested using Tissue Digestion Buffer (1 mg/mL Collagenase IV + 50 µg/mL DNAse I in AIM V medium) for 45 minutes while shaking at 500 RPM on an orbital shaker in a 37°C incubator. The samples were passed through a 70 µm filter, then washed in PBS before staining for flow cytometry. Samples were stained with Fixable Viability Dye 780 to label dead cells for 15 minutes. Fc receptors were blocked using a blocking buffer: PBS + 10% FBS + FcX anti-mouse CD16/32 for 15 minutes. Samples were stained for 30 min, using the following anti-mouse antibodies: CD4-Alexa Fluor 647, CD8-PerCP, TCRb-BV605, CD11b-PE, CD19-BV480, CD45-BV421, CD161 (NK1.1)-PE-Fire700, CD185 (CXCR5)-BV785, and F4/80-Alexa Fluor 594. All staining steps for flow cytometry were performed at 4°C, covered to protect from light. Samples were collected on a Novocyte Quanteon flow cytometer (Agilent Technologies) using NovoExpress software and analyzed using FlowJo software (BD).

## Data Availability

All figures report primary data values. Data will be made available upon reasonable request, in line with institutional guidelines. No novel code tools were required for data analysis.

## SUPPLEMENTAL MATERIAL

Figure S1 mbio.02254-25-s0001.tif LNP molecular structures and organoid responses.

Figure S2 mbio.02254-25-s0002.tif Representative flow cytometry gating.

Figure S3 mbio.02254-25-s0003.tif Tonsil, spleen, and PBMC responses to mRNA LNP stimulation.

Table S1 mbio.02254-25-s0004.tif Donor source and information.

ASM does not own the copyrights to Supplemental Material that may be linked to, or accessed through, an article. The authors have granted ASM a non-exclusive, world-wide license to publish the Supplemental Material files. Please contact the corresponding author directly for reuse.
