# Supplementary figures and images for: Human CD4 T cells are a functional target for lipid nanoparticle-based mRNA vaccines
Source: mBio. 2025 Sep 22;16(11):e02254-25. doi: 10.1128/mbio.02254-25 (PMC12607866; doi:10.1128/mbio.02254-25)

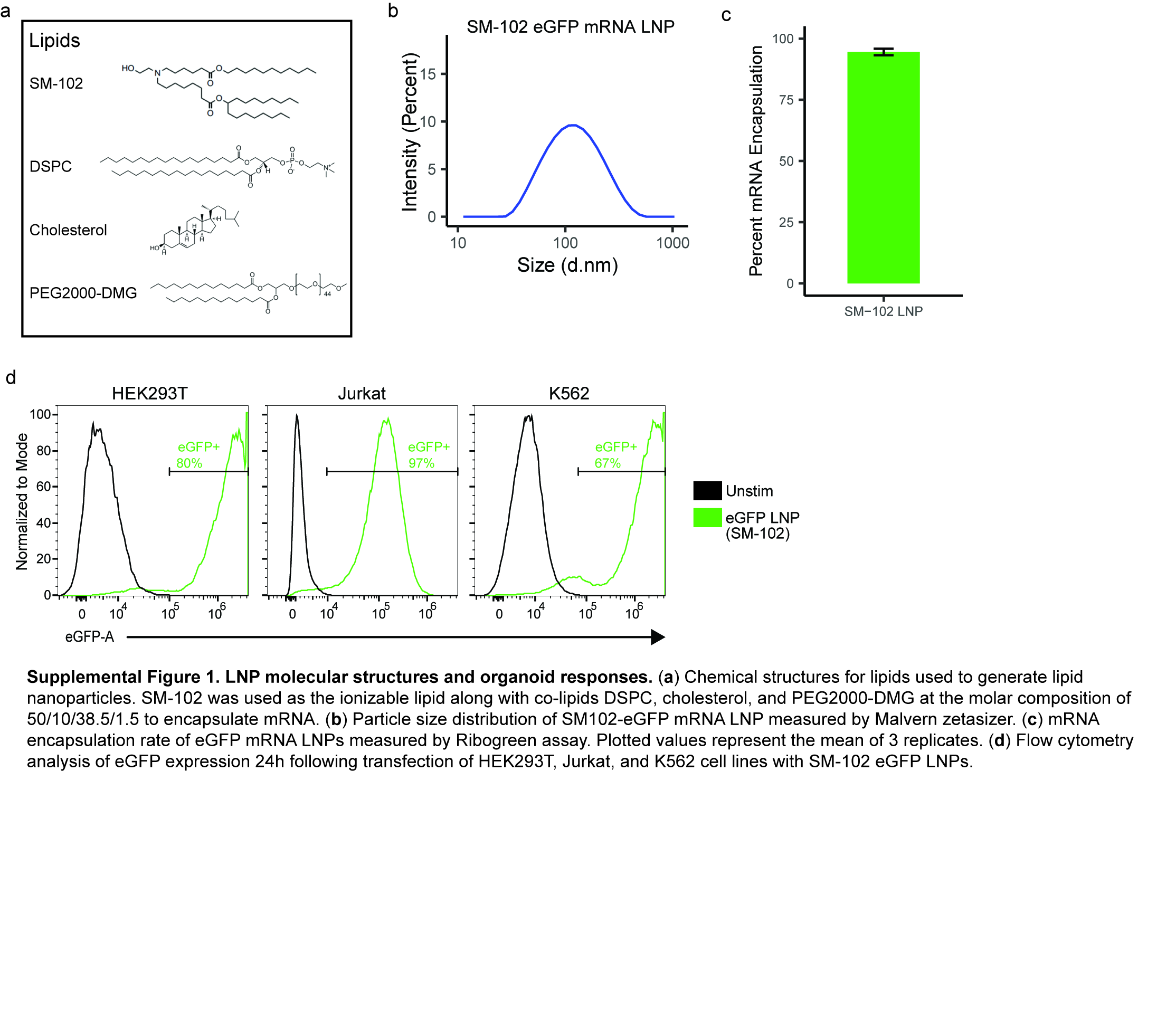

Supplement: Figure S1 — LNP molecular structures and organoid responses. [file mbio.02254-25-s0001.tif]

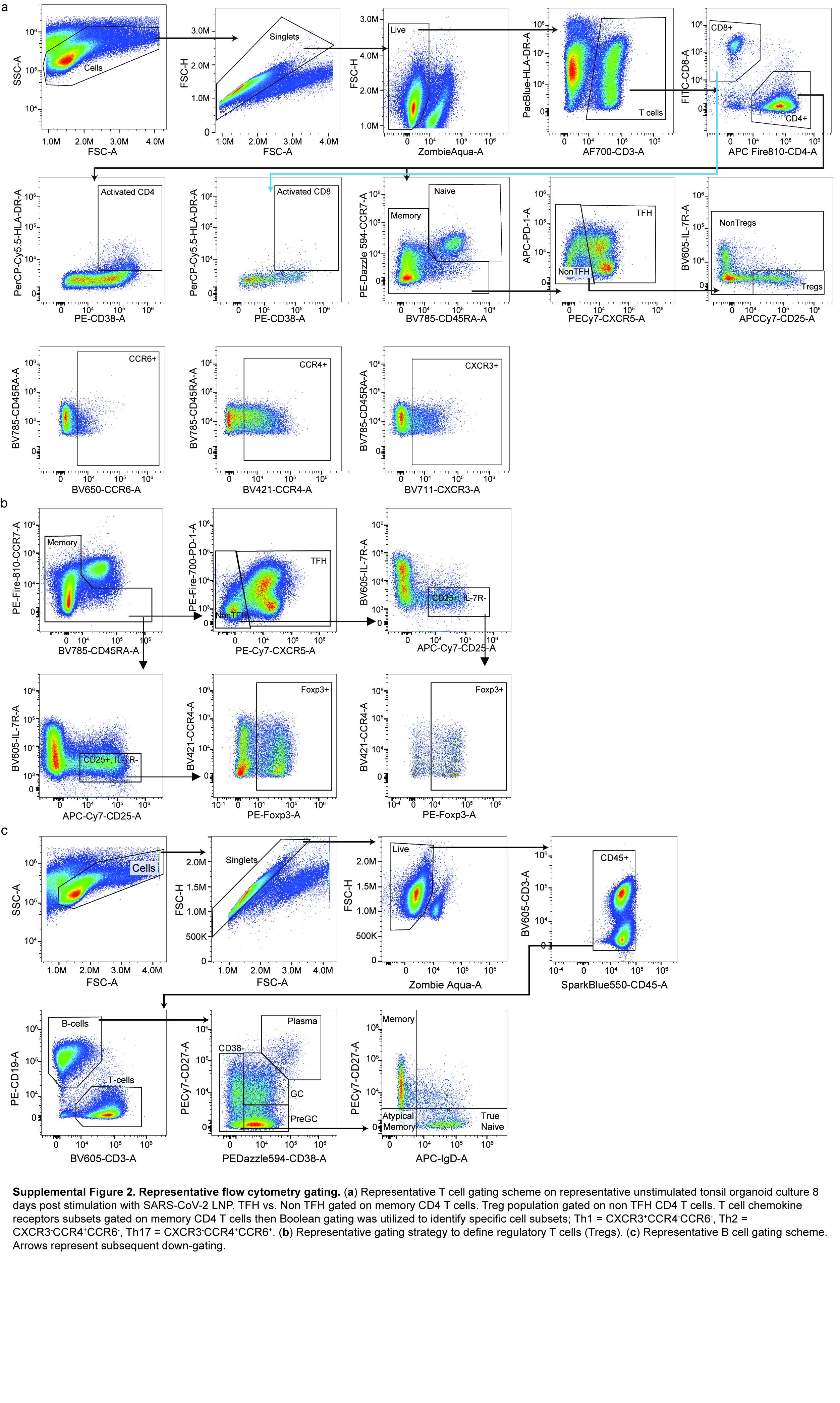

Supplement: Figure S2 — Representative flow cytometry gating. [file mbio.02254-25-s0002.tif]

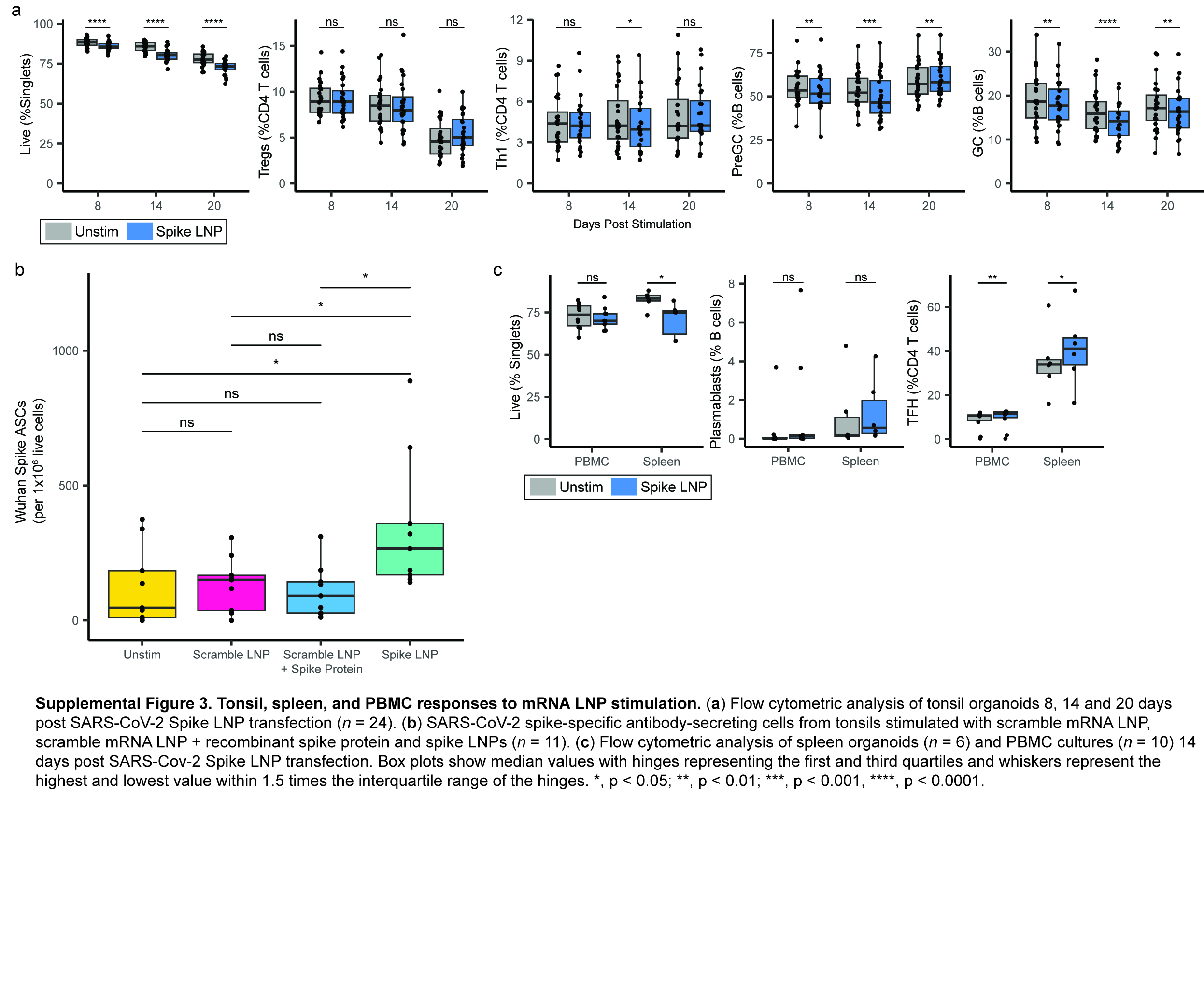

Supplement: Figure S3 — Tonsil, spleen, and PBMC responses to mRNA LNP stimulation. [file mbio.02254-25-s0003.tif]
